# Impact of embryo transfer strategies on children health outcomes: a retrospective national cohort study in Taiwan

**DOI:** 10.3389/fendo.2025.1630293

**Published:** 2025-07-11

**Authors:** Chih-Ting Chang, Shih-Feng Weng, Hui-Yu Chuang, Chia-Yi Hsu, Eing-Mei Tsai

**Affiliations:** ^1^ NUWA Fertility Center, Taipei, Taiwan; ^2^ Department of Healthcare Administration and Medical Informatics, Kaohsiung Medical University, Kaohsiung, Taiwan; ^3^ Center for Medical Informatics and Statistics, Office of Research and Development, Kaohsiung Medical University, Kaohsiung, Taiwan; ^4^ Center for Big Data Research, Kaohsiung Medical University, Kaohsiung, Taiwan; ^5^ Department of Obstetrics and Gynecology, Kaohsiung Medical University Hospital, Kaohsiung, Taiwan; ^6^ Graduate Institute of Medicine, College of Medicine, Kaohsiung Medical University, Kaohsiung, Taiwan

**Keywords:** assisted reproductive technology (ART), embryo transfer, frozen embryo transfer (FET), child health and development, fresh embryo transfer (fET), perinatal outcomes

## Abstract

**Objective:**

To investigate the impact of different assisted reproductive technology (ART) embryo transfer strategies on neonatal and early childhood health outcomes, focusing on fresh versus frozen transfers and cleavage versus blastocyst stages.

**Design:**

Retrospective cohort study analyzing data from Taiwan’s national assisted reproduction database (2013–2017).

**Methods:**

The study included 10,803 ART-conceived singleton births and 894,615 naturally conceived singletons. ART groups were categorized by embryo transfer type: fresh cleavage stage, fresh blastocyst, frozen cleavage stage, and frozen blastocyst. Maternal, paternal, and perinatal outcomes were adjusted using inverse probability of treatment weighting (IPTW). Outcomes included major and minor diseases in offspring, such as ADHD, developmental delays, atopic dermatitis, and respiratory diseases.

**Results:**

ART-conceived children had higher rates of major and minor health conditions compared to naturally conceived peers, particularly preterm birth, ADHD, and developmental delay. No significant differences were observed in major disease incidence between frozen and fresh transfers or cleavage and blastocyst stages. The elevated risks in ART-conceived children may reflect the influence of underlying parental infertility rather than ART procedures alone.

**Conclusion:**

While ART is linked to increased risks of certain adverse health outcomes, the choice between embryo transfer strategies has minimal impact on neonatal or early childhood health. These findings underscore the need to optimize ART protocols and perinatal care while addressing the role of parental infertility in shaping offspring health.

## Introduction

Since the birth of the first *in vitro* fertilization (IVF)-conceived child in 1978 (1), assisted reproductive technology (ART) has enabled the conception of over 10 million children worldwide (2). However, substantial research has raised concerns about the health outcomes of ART-conceived offspring, highlighting risks such as cardiovascular, musculoskeletal, chromosomal defects, urogenital diseases, and cancers (3–5).

More recent studies have suggested potential links between specific ART procedures, like intracytoplasmic sperm injection (ICSI), and neurodevelopmental disorders, bringing into question the safety of these techniques (6). Globally, the number of children born following ART with frozen- thawed embryo transfer (FET) has surpassed those born through fresh embryo transfer in many regions (7, 8). This trend is further driven by the increasing adoption of extended embryo culture, particularly under single embryo transfer policies (9). However, both FET and extended embryo culture may induce epigenetic changes, influenced by variables such as temperature, gas concentration, and pH fluctuations during the procedures. These changes could have significant implications for child health, particularly concerning increased birth weights and a heightened risk of large-for-gestational-age (LGA) outcomes after frozen embryo transfers (10–14). Consequently, it is essential to verify the long-term health outcomes for children conceived through FET or extended embryo culture.

In response to these concerns, this study seeks to investigate the impact of different embryo transfer strategies on the health outcomes of children conceived through IVF. Utilizing a comprehensive dataset that includes various patient demographics and medical variables, the study aims to clarify potential associations between embryo transfer techniques (including FET and extended embryo culture) and singleton health outcomes up to early childhood (2–5 years of age) in Taiwan. The findings will provide essential insights into the implications of ART procedures on child health, informing both clinical practice and policy decisions surrounding IVF and ART.

## Materials and methods

This retrospective cohort study was conducted in Taiwan and approved by the institutional review board of Kaohsiung Medical University Chung-Ho Memorial Hospital, IRB-No. KMUHIRB-E(I)-20210222, which waived the requirement for informed consent because the data were encrypted and deidentified.

More than 99% of the citizens of Taiwan have participated in the National Health Insurance program since 1995, and the national population registry data set is linked to the national ART and birth certification data set. Couples who entered the IVF treatment in Taiwan have been completely recorded in the Taiwan national ART database. The ART database in Taiwan was established in the year 1998. It collects case data of individuals who undergo assisted reproduction procedures at the respective reproductive institutions, excluding assisted insemination between spouses. Medical information in the national population registry data set is recorded at the time of visit to outpatient public or private clinics. The databases have undergone de-identification processes, including the removal of directly identifiable fields such as names and addresses. Sensitive fields such as identification numbers, institution codes, insurance policy unit codes, tax identification numbers, dates of birth, medical dates, and admission dates have been masked to comply with the strong data protection standards of FIPS 140–2 Level 3 international security standards. The related data can only be used within the independent operating area set up by the authority, and any disclosed statistical results are carefully reviewed to ensure that there is no possibility of identifying specific individuals through the data application or disclosure methods.

### Participants flow chart

**Figure 1:**
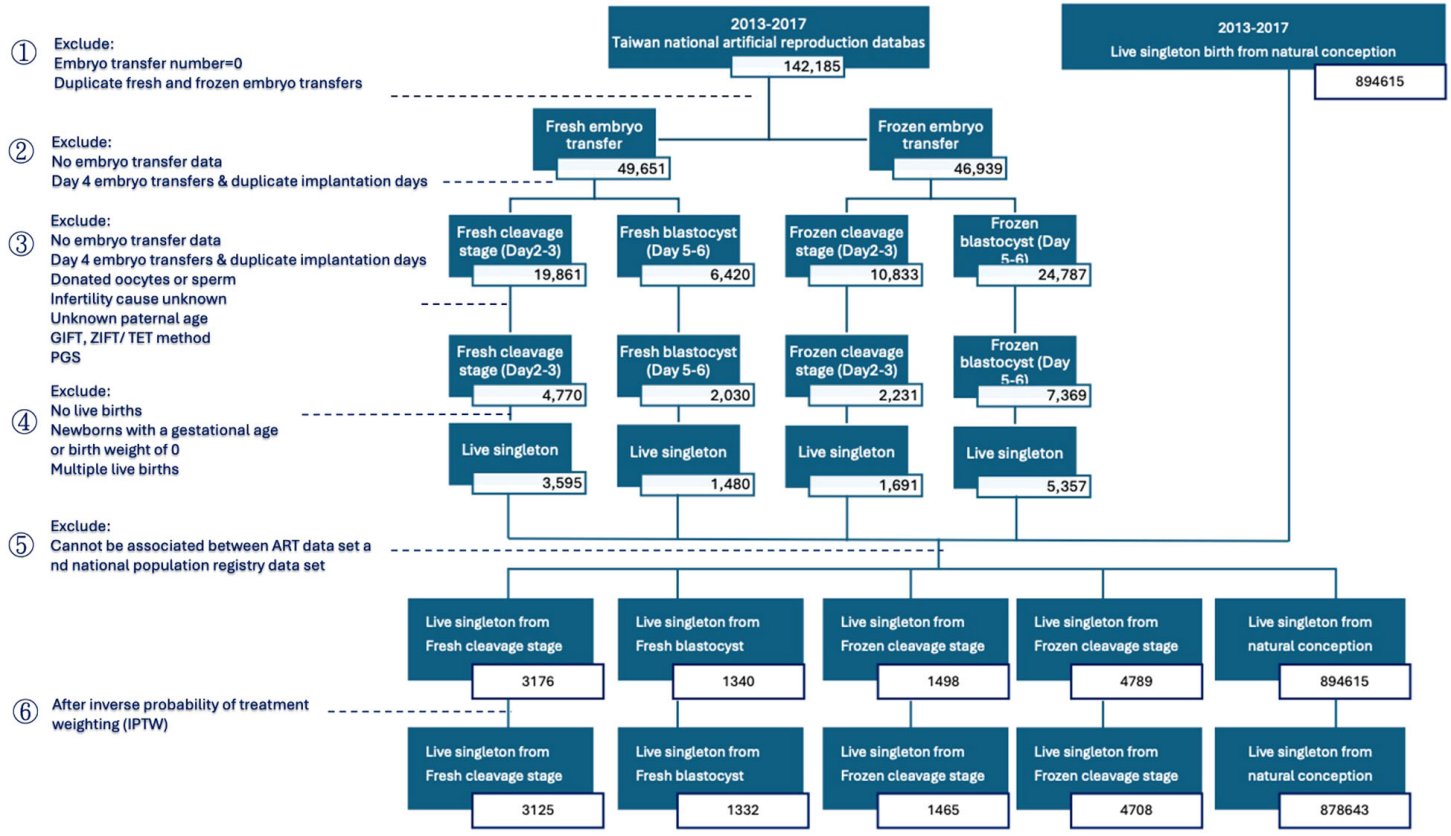
Flowchart illustrating the process of refining the study population, including application of inclusion and exclusion criteria.

The flowchart details the process of refining data from the Taiwan national assisted reproduction database between January 1, 2013, and December 31, 2017 to study live singleton births from ART and natural conception. The initial dataset comprised 142,185 ART records and 894,615 live singleton births from natural conception. The first step involved excluding records with an embryo transfer number of zero and duplicate fresh and frozen embryo transfers, resulting in 49,651 fresh embryo transfers and 46,939 frozen embryo transfers. Subsequent exclusions targeted records lacking embryo transfer data, day 4 embryo transfers, and duplicate implantation days. Further exclusions eliminated records involving donated oocytes or sperm, unknown infertility causes, unknown paternal age, and specific ART methods such as gamete intrafallopian transfer (GIFT), zygote intrafallopian transfer/tubal embryo transfer (ZIFT/TET), as well as those with preimplantation genetic screening (PGS). The analysis then focused on excluding records without live births, gestational age or birth weight of zero, and multiple live births, yielding 3,595 live singleton births from fresh cleavage stages, 1,480 from fresh blastocysts, 1,691 from frozen cleavage stages, and 5,357 from frozen blastocysts. Another exclusion phase removed records that couldn’t be associated with the national population registry, refining the data to 3,176 live singleton births from fresh cleavage stages (Day 2-3), 1,340 from fresh blastocysts (Day 5-6), 1,498 from frozen cleavage stages, and 4,789 from frozen blastocysts. Following inverse probability of treatment weighting (IPTW), the final dataset consisted of 3,125 live singleton births from fresh cleavage stages, 1,332 from fresh blastocysts, 1,465 from frozen cleavage stages, and 4708 from frozen blastocysts, compared against 878,643 singleton births from natural conception (**Figure 1**).

The basic information for both natural and ART conceptions was identified, including maternal age, paternal age, maternal risk factors during pregnancy (hypertensive disorder, diabetes, gestational diabetes), maternal complications during labor (prolonged premature rupture of membrane >12 hours, placental abruption, placenta previa, postpartum hemorrhage).

### Exposure

We evaluated neonatal and child health outcomes in offspring based on different embryo transfer strategies, including frozen versus fresh embryo transfer and the use of extended embryo culture. We categorized outcomes into major diseases—such as birth injuries, chromosomal abnormalities, cardiac septum malformations, orofacial clefts, vesicoureteral reflux, ADHD, developmental delays, Leukemia, melanoma, and malignant neoplasms of skin—and minor diseases, including otitis media, torticollis, heart murmurs, newborn respiratory distress, recurrent upper respiratory infections, croup, colic, jaundice, urinary tract infections, pyelonephritis, atopic dermatitis, asthma, and eczema. Major malformations were defined as disorders that caused functional impairment or required surgical correction.

### Statistical analysis

Baseline characteristics of the study population were compared across the five groups using the chi-square test. These characteristics included maternal age, paternal age, risk factor during pregnancy (including pregnancy induced hypertension or chronic hypertension, Gestational DM or DM, unhealthy lifestyle, placenta previa, placenta abruption, preterm premature rupture of membrane, and postpartum hemorrhage).

To reduce potential confounding arising from differences in the distribution of measured baseline characteristics among groups in this observational study, we applied inverse probability of treatment weighting (IPTW). Propensity scores were calculated using multinomial logistic regression to balance the baseline characteristics across groups. Maternal age, along with other covariates, was included in the propensity score model to minimize age-related confounding between the ART and natural conception groups.

Subsequently, we weighted each group by the inverse of the probability of their treatment allocation and created the pseudo data set (15). A weighted χ2 test was utilized to assess the balance of baseline characteristics among the groups. After IPTW, the difference in neonatal outcomes (such as gestational age, newborn body weight, route of delivery, and Apgar score at 1 and 5 minutes)), and child health outcomes among the groups were also estimated using weighted χ2 test.

To compare neonatal outcomes between the groups (e.g., Fresh blastocyst versus Fresh cleavage stage, Frozen blastocyst versus Frozen cleavage stage, Frozen cleavage stage versus Fresh cleavage stage, Frozen blastocyst versus Fresh blastocyst, Fresh cleavage stage versus Natural conception, Fresh blastocyst versus Natural conception, Frozen cleavage stage versus Natural conception, and Frozen blastocyst versus Natural conception), multivariate logistic regression analyses were performed. These analyses adjusted for confounding variables (pregnancy induced hypertension or chronic hypertension, Gestational DM or DM, unhealthy lifestyle, placenta previa, placenta abruption, preterm premature rupture of membrane, and postpartum hemorrhage) and used IPTW to calculate the odds ratios (OR).

For the longitudinal children health outcomes, the risk of major and minor disease between the groups (e.g., Fresh blastocyst versus Fresh cleavage stage, Frozen blastocyst versus Frozen cleavage stage, Frozen cleavage stage versus Fresh cleavage stage, and Frozen blastocyst versus Fresh blastocyst) was estimated using hazard ratios (HR) through Cox Proportional Hazards regression, adjusting for (pregnancy induced hypertension or chronic hypertension, Gestational DM or DM, unhealthy lifestyle, placenta previa, placenta abruption, preterm premature rupture of membrane, and postpartum hemorrhage) confounders.

For the descriptive statistical analysis, a p-value of less than 0.05 (two-tailed) was considered statistically significant. However, due to the large number of hypothesis tests performed (multiple testing issue), a more stringent p-value threshold of 0.005 with Bonferroni correction (significance level/number of the tests) was considered significant. All analyses were performed using SAS 10(SAS Institute Inc., Cary, NC).

## Outcome

**Table 1** presents the Characteristics of the Study Population. The final cohort study included 10,803 eligible singletons born through ART embryo transfer and 894,615 through natural conception.

**Table 1:** Characteristics of the study population.

	Before IPTW											After IPTW										
	Fresh cleavage stage (Day 2-3) (N=3,176)		Fresh blastocyst (Day 5-6) (N=1,340)		Frozen cleavage stage (Day 2-3) (N=1,498)		Frozen blastocyst (Day 5-6) (N=4,789)		Natural conception (N=894,615)		p-value	Fresh cleavage stage (Day 2-3) (N=3,125)		Fresh blastocyst (Day 5-6) (N=1,332)		Frozen cleavage stage (Day 2-3) (N=1,465)		Frozen blastocyst (Day 5-6) (N=4,708)		Natural conception (N=878,643)		p-value
	N	%	N	%	N	%	N	%	N	%		N	%	N	%	N	%	N	%	N	%	
**Maternal age**																						
<30y	165	5.20	118	8.81	82	5.47	415	8.67	273,235	30.54	<.0001	941	30.12	409	30.69	438	29.92	1405	29.85	270687	30.81	0.9534
30-35y	975	30.70	554	41.34	456	30.44	1,779	37.15	365,731	40.88		1303	41.70	549	41.21	611	41.71	1959	41.60	365006	41.54	
35-40y	1,601	50.41	581	43.36	740	49.40	2,141	44.71	206,950	23.13		752	24.08	321	24.12	356	24.31	1151	24.45	209441	23.84	
>40y	435	13.70	87	6.49	220	14.69	454	9.48	32,720	3.66		128	4.09	53	3.98	59	4.03	193	4.10	33510	3.81	
**Paternal age**																						
≦40y	2,294	72.23	1,031	76.94	1,073	71.63	3,711	77.49	785,419	87.79	<.0001	2748	87.94	1179	88.51	1275	87.06	4129	87.69	783888	89.22	<.0001
>40y	882	27.77	309	23.06	425	28.37	1,078	22.51	93,217	10.42		377	12.05	153	11.49	189	12.91	580	12.31	94755	10.78	
**Risk factor during pregnancy**																						
Pregnancy induced hypertension or chronic hypertension	124	3.90	36	2.69	73	4.87	191	3.99	11,611	1.30	<.0001	52	1.67	18	1.34	27	1.85	71	1.51	11893	1.35	0.2023
Gestational DM or DM	193	6.08	64	4.78	90	6.01	223	4.66	17,864	2.00	<.0001	72	2.29	31	2.34	38	2.58	118	2.52	18215	2.07	0.1112
Unhealthy life style (smoking, drinking, and drugs )	471.00										0.6021	3	0.11	0	0.00	0	0.00	1	0.03	465	0.05	0.3992
Placenta previa	86	2.71	46	3.43	25	1.67	116	2.42	6,459	0.72	<.0001	30	0.97	11	0.84	14	0.93	46	0.97	6655	0.76	0.2518
Placenta abruption	33	1.04	11	0.82	12	0.80	16	0.33	3,100	0.35	<.0001	13	0.41	5	0.37	11	0.72	18	0.39	3135	0.36	0.2194
Preterm premature rupture of membrane (PPROM)	96	3.02	42	3.13	59	3.94	162	3.38	15,204	1.70	<.0001	71	2.27	19	1.42	26	1.77	93	1.97	15375	1.75	0.1297
Postpartum hemorrhage	35	1.10	25	1.87	52	3.47	90	1.88	4,046	0.45	<.0001	17	0.54	6	0.46	8	0.57	30	0.63	4201	0.48	0.5714

The red text indicates variables with statistically significant P values.

Before applying Inverse Probability of Treatment Weighting (IPTW), significant differences were observed between the groups. In both fresh and frozen embryo transfer scenarios, the blastocyst groups had a higher proportion of younger mothers and fathers, while the cleavage stage groups had a higher percentage of older individuals. Among women under 35 years old, natural conception had the highest proportion, followed by the blastocyst stage, and then the cleavage stage (50.15% in fresh blastocyst vs. 35.9% in fresh cleavage stage; 45.82% in frozen blastocyst vs. 35.91% in frozen cleavage stage; 71.42% in natural conception). Conversely, for women aged 40 and above, the cleavage stage had the highest proportion, followed by the blastocyst stage, and then natural conception (13.7% in fresh cleavage stage vs. 6.49% in fresh blastocyst stage; 14.69% in frozen cleavage stage vs. 9.48% in frozen blastocyst stage, 3.66% in natural conception). The same pattern was observed for paternal age: fathers under 40 years old were most prevalent in natural conception, followed by the blastocyst stage, and then the cleavage stage (76.94% in fresh blastocyst vs. 72.23% in fresh cleavage stage; 77.49% in frozen blastocyst vs. 71.63% in frozen cleavage stage, 87.79% in natural conception).

In terms of pregnancy risk factors, pregnancy-induced hypertension was more common in the frozen groups (3.99% in frozen blastocyst, 4.87% in frozen cleavage stage) compared to the fresh groups (2.69% in fresh blastocyst, 3.9% in fresh cleavage stage) and natural conception (1.30%). Gestational diabetes mellitus was more prevalent in the cleavage stage groups (6.08% in fresh cleavage vs. 4.78% in fresh blastocyst; 6.01% in frozen cleavage vs. 4.66% in frozen blastocyst, 2.00% in natural conception). Additionally, gestational complications, such as placenta previa, placenta abruption, and preterm premature rupture of membranes (PPROM), were more frequent in ART groups compared to natural conception.

After implementing IPTW, significant disparities in maternal age and pregnancy risk factors were no longer observed. However, paternal age remained younger in the natural conception group. The distribution of individuals across different groups after IPTW adjustment was as follows: 3,125 for fresh cleavage stage, 1,332 for fresh blastocyst, 1,465 for frozen cleavage stage, 4,708 for frozen blastocyst, and 878,643 for natural conception. The Standardized Mean Differences (SMD) for these variables indicated that balance in maternal age and pregnancy risk factor was achieved after IPTW adjustment.

**Table 2** presents the perinatal outcomes and incidence rates of various major and minor diseases in children conceived via different ART methods and natural conception. The table compares fresh cleavage stage transfer (Day 2-3), frozen cleavage stage transfer (Day 5-6), fresh blastocyst transfer (Day 5-6), frozen blastocyst transfer (Day 5-6), and natural conception. Each disease is listed with its corresponding ICD-10 and ICD-9 codes, the number of cases (N), and the percentage (%).

**Table 2:** Children health outcomes.

	ICD10	ICD9	Fresh cleavage stage (Day 2-3) (N=3,125)		Frozen cleavage stage (Day 5-6) (N=1,465)		Fresh blastocyst (Day 2-3) (N=1,232)		Frozen blastocyst (Day 5-6) (N=4,708)		Natural conception (N=878,643)		p-value
			N	%	N	%	N	%	N	%	N	%	
**Birth outcomes**													
Birth injury	P00-P15	760-767	35	1.11	22	1.52	22	1.82	73	1.55	6,102	0.69	<.0001
Preterm labor (week<37)													
Birth weight <2500g			349	11.17	145	10.89	131	8.93	383	8.13	56,924	6.48	
Birth weight ≧4000g			24	0.77	20	1.46	23	1.57	93	1.98	12,282	1.40	
SGA			427	13.67	179	13.44	141	9.62	402	8.53	78,406	8.92	<.0001
LGA			153	4.90	82	6.15	116	7.94	468	9.94	55,959	6.37	<.0001
**Major disease**													
Chromosomal abnormalities	Q90-Q99	758	6	0.21	2	0.10	1	0.09	4	0.09	599	0.07	0.0479
Malformation of cardiac septum	Q20,Q21	745	87	2.79	42	2.86	50	4.06	163	3.47	16,068	1.83	<.0001
Orofacial cleft	Q35, Q36, Q37	749	2	0.05	3	0.21	1	0.09	6	0.13	1,375	0.16	0.5259
Vesicoureteral reflux	N13.7, N13.9	593.7	16	0.50	6	0.40	10	0.82	11	0.23	2,223	0.25	0.0002
ADHD	F90	314	5	0.17	3	0.23	2	0.15	5	0.11	3,387	0.39	0.0027
Developmental delay	F80-82	315	35	1.12	21	1.43	17	1.34	59	1.26	14,091	1.60	0.0520
Leukemia	C91-C95	203-208	0	0.00	0	0.00	0	0.00	1	0.03	286	0.03	0.8233
Melanoma	C43	172	0	0.00	0	0.00	0	0.00	0	0.00	3	0.00	0.9998
malignant neoplasms of skin	C44	173	0	0.00	0	0.00	0	0.00	0	0.00	8	0.00	0.9989
**Minor disease**													
Otitis media	H65	381.0-381.4	52	1.65	28	1.93	28	2.27	65	1.37	20,313	2.31	<.0001
Torticollis	G24.3, M43.6, R29.891	333.83,723.5	8	0.26	6	0.38	9	0.73	14	0.30	1,652	0.19	0.0001
Newborn respiratory distress	P20-P28	769-770	29	0.93	21	1.45	22	1.78	60	1.27	5,454	0.62	<.0001
Recurrent upper respiratory infection (>5times)	J06	465, 466	1,544	49.39	607	41.46	581	47.16	2,025	43.01	542,100	61.70	<.0001
Croup (obstructive laryngitis)	J05	464	107	3.42	44	3.02	32	2.56	189	4.01	53,447	6.08	<.0001
Colic	R10.83	789	7	0.24	7	0.48	1	0.11	11	0.24	10,212	1.16	<.0001
Jaundice	P58-P59	774.0-774.6	19	0.60	9	0.62	10	0.79	38	0.80	5,943	0.68	0.8331
Urinary tract infection	N30.0, P39.3	599	16	0.52	1	0.06	7	0.57	9	0.18	23,863	2.72	<.0001
Pyelonephritis	N11, N16	590.1, 590.8	0	0.01	1	0.04	1	0.09	2	0.04	3,779	0.43	<.0001
Atopic dermatitis	L209, L2089	691.8	218	6.96	90	6.15	62	5.05	310	6.58	52,441	5.97	0.0133
Asthma	J45	493.0, 493.1, 493.9	137	4.39	54	3.66	54	4.39	225	4.79	75,359	8.58	<.0001
Eczema and dermatitis	L20-L30	690-692.6	580	18.57	227	15.49	260	21.11	800	16.99	161,723	18.41	0.0035

The red text indicates variables with statistically significant P values.

**Table 3** compare four different embryo transfer methods and stages (fresh blastocyst, frozen blastocyst, frozen cleavage, and fresh cleavage) to natural conception, while **Table 4** compare these four methods against each other. Odds ratios (ORs) are used to assess perinatal outcomes, while hazard ratios (HRs) indicate differences in disease incidence rates. A p-value of < 0.005 is considered statistically significant. When compared to natural conception (**Table 3**), the use of ART is associated with an increased risk for preterm labor, low birth weight, and several major and minor diseases. However, no significant differences were observed for orofacial cleft, croup, and jaundice. Notably, the HR for attention-deficit/hyperactivity disorder (ADHD) is markedly elevated in ART-conceived children, particularly in the fresh blastocyst group, which demonstrated the highest HR (HR 19.49, 95% CI 6.71-56.61, p<0.0001) relative to natural conception. Conversely, the incidence of genitourinary conditions, such as urinary tract infections and pyelonephritis, is lower in the ART groups compared to those conceived naturally. In this study, melanoma, malignant neoplasms of the skin, and leukemia were rare outcomes of interest. Across all groups, the number of cases for these conditions was zero, except for a single leukemia case observed in the frozen blastocyst group. Due to the absence of events in most groups, it was not possible to calculate hazard ratios (HRs) using Cox proportional hazards regression.

**Table 3:** Children health outcomes compared to natural conception.

	Fresh cleavage stageversusNatural Conception				Fresh blastocystversusNatural Conception				Frozen blastocyst versus Natural Conception				Frozen cleavage stage versus Natural Conception			
Birth outcomes	OR	(95% CI)		*P-value*	OR	(95% CI)		*P-value*	OR	(95% CI)		*P-value*	OR	(95% CI)		*P-value*
Birth injury	1.54	1.10	2.15	0.0112	2.17	1.43	3.29	0.0003*	2.61	1.72	3.95	<.0001*	2.32	1.84	2.92	<.0001*
Preterm labor (week<37)	1.671	1.489	1.874	<.0001*	1.595	1.331	1.911	<.0001*	1.546	1.300	1.837	<.0001*	1.579	1.434	1.738	<.0001*
Birth weight <2500g	1.794	1.601	2.012	<.0001*	1.818	1.525	2.167	<.0001*	1.379	1.147	1.658	0.0006*	1.268	1.139	1.411	<.0001*
Birth weight ≧4000g	0.540	0.361	0.807	0.0027*	1.040	0.664	1.628	0.8647	1.101	0.728	1.665	0.6490	1.402	1.141	1.723	0.0013*
SGA	1.618	1.460	1.793	<.0001*	1.594	1.361	1.868	<.0001*	1.080	0.907	1.286	0.3886	0.956	0.863	1.060	0.3946
LGA	0.750	0.637	0.882	0.0005*	0.958	0.765	1.198	0.7053	1.251	1.034	1.514	0.0211	1.607	1.459	1.769	<.0001*
	
**Major disease**	**HR**	**(95% CI)**		***P-value***	**HR**	**(95% CI)**		***P-value***	**HR**	**(95% CI)**		***P-value***	**HR**	**(95% CI)**		***P-value***
Chromosomal abnormalities	5.16	2.38	11.19	<.0001*	3.13	0.64	15.38	0.1602	2.14	0.33	14.09	0.4284	2.71	1.07	6.88	0.0361
Malformation of cardiac septum	1.91	1.55	2.36	<.0001*	2.24	1.65	3.03	<.0001*	2.68	2.03	3.54	<.0001*	2.63	2.26	3.07	<.0001*
Orofacial cleft	0.35	0.07	1.66	0.1857	1.51	0.49	4.66	0.4699	0.58	0.09	3.79	0.5672	0.94	0.42	2.07	0.8682
Vesicoureteral reflux	2.46	1.49	4.03	0.0004 *	2.13	0.94	4.80	0.0695	3.81	2.06	7.07	<.0001*	1.21	0.67	2.20	0.5253
ADHD	6.95	2.96	16.30	<.0001*	19.49	6.71	56.61	<.0001*	7.25	1.69	31.20	0.0078	7.20	3.00	17.29	<.0001*
Developmental delay	2.84	2.04	3.96	<.0001*	6.30	4.10	9.67	<.0001*	3.67	2.26	5.94	<.0001*	4.63	3.59	5.98	<.0001*
Leukemia	--	--	--	0.9625	2.20	0.09	54.01	0.6299	--	--	--	0.9765	2.09	0.36	12.08	0.4087
Melanoma	--	--	--	0.9997	--	--	--	0.9999	--	--	--	0.9998	--	--	--	0.9997
malignant neoplasms of skin	--	--	--	0.9991	--	--	--	0.9995	--	--	--	0.9995	--	--	--	0.9990
	
**Minor disease**	**HR**	**(95% CI)**		***P-value***	**HR**	**(95% CI)**		***P-value***	**HR**	**(95% CI)**		***P-value***	**HR**	**(95% CI)**		***P-value***
Otitis media	2.03	1.54	2.67	<.0001*	3.55	2.46	5.13	<.0001*	2.85	1.97	4.13	<.0001*	2.20	1.72	2.81	<.0001*
Torticollis	1.72	0.86	3.43	0.1253	2.86	1.25	6.55	0.0127	4.63	2.41	8.92	<.0001*	2.18	1.30	3.68	0.0034 *
Heart murmur	2.35	1.63	3.39	<.0001*	1.67	0.83	3.39	0.1516	1.42	0.68	2.99	0.3559	2.24	1.60	3.11	<.0001*
Newborn respiratory distress	1.44	1.00	2.08	0.0488	2.30	1.50	3.52	0.0001 *	2.82	1.86	4.29	<.0001*	2.11	1.63	2.72	<.0001*
Recurrent upper respiratory infection	1.35	1.29	1.42	<.0001*	1.51	1.39	1.63	<.0001*	1.27	1.17	1.38	<.0001*	1.43	1.37	1.50	<.0001*
Croup (obstructive laryngitis)	0.85	0.71	1.03	0.0976	1.00	0.75	1.35	0.9899	0.63	0.44	0.89	0.0090	1.21	1.05	1.39	0.0103
Colic	0.31	0.15	0.64	0.0015 *	0.79	0.38	1.66	0.5354	0.14	0.03	0.76	0.0226	0.37	0.21	0.66	0.0008*
Jaundice	0.91	0.58	1.43	0.6706	0.95	0.50	1.83	0.8877	1.14	0.60	2.13	0.6938	1.25	0.91	1.72	0.1756
Urinary tract infection	0.21	0.13	0.35	<.0001*	0.03	0.00	0.22	0.0006 *	0.22	0.11	0.47	<.0001*	0.08	0.04	0.16	<.0001*
Pyelonephritis	0.02	0.00	0.95	0.0469	0.11	0.01	1.39	0.0885	0.22	0.03	1.38	0.1059	0.11	0.03	0.44	0.0022*
Atopic dermatitis	1.82	1.59	2.08	<.0001*	2.04	1.66	2.50	<.0001*	1.29	1.01	1.66	0.0431	2.04	1.82	2.28	<.0001*
Asthma	1.73	1.47	2.05	<.0001*	2.28	1.75	2.98	<.0001*	1.82	1.40	2.38	<.0001*	2.56	2.24	2.92	<.0001*
Eczema and dermatitis	1.51	1.39	1.64	<.0001*	1.57	1.38	1.79	<.0001*	1.69	1.50	1.91	<.0001*	1.62	1.51	1.73	<.0001*

*p<0.005.

Red text represents hazard ratios (HR) greater than 1 with statistical significance, while blue text indicates HRs less than 1 with statistical significance.

**Table 4:** Children health outcomes compared in different transfer methods.

	Fresh blastocystversusFresh cleavage stage				Frozen blastocyst versusFrozen cleavage stage				Frozen cleavage stage versus Fresh cleavage stage				Frozen blastocyst versus Fresh blastocyst			
Birth outcomes	OR	(95% CI)		*P-value*	OR	(95% CI)		*P-value*	OR	(95% CI)		*P-value*	OR	(95% CI)		*P-value*
Birth injury	1.54	1.10	2.15	0.0112	2.17	1.43	3.29	0.0003*	2.61	1.72	3.95	<.0001*	2.32	1.84	2.92	<.0001*
Preterm labor (week<37)	0.955	0.771	1.182	0.6702	1.022	0.839	1.245	0.8320	0.925	0.752	1.138	0.4626	0.990	0.807	1.214	0.9220
Birth weight <2500g	1.013	0.822	1.249	0.9042	0.918	0.742	1.136	0.4306	0.769	0.619	0.954	0.0172	0.696	0.567	0.855	0.0005*
Birth weight ≧4000g	1.926	1.055	3.517	0.0328	1.274	0.803	2.021	0.3043	2.039	1.146	3.630	0.0154	1.348	0.824	2.207	0.2343
SGA	0.986	0.816	1.190	0.8807	0.886	0.723	1.084	0.2394	0.668	0.545	0.817	<.0001*	0.600	0.497	0.724	<.0001*
LGA	1.278	0.968	1.686	0.0832	1.284	1.038	1.589	0.0213	1.669	1.299	2.144	<.0001*	1.678	1.315	2.141	<.0001*
	
**Major disease**	**HR**	**(95% CI)**		***P-value***	**HR**	**(95% CI)**		***P-value***	**HR**	**(95% CI)**		***P-value***	**HR**	**(95% CI)**		***P-value***
Birth injury	1.41	0.83	2.40	0.2069	0.89	0.55	1.43	0.6250	1.69	1.00	2.88	0.0520	1.20	0.67	2.16	0.5387
Chromosomal abnormalities	0.61	0.10	3.54	0.5788	1.27	0.16	10.31	0.8258	0.42	0.05	3.17	0.3965	0.68	0.06	8.03	0.7626
Malformation of cardiac septum	1.17	0.81	1.69	0.4017	0.98	0.72	1.35	0.9167	1.40	0.99	1.98	0.0572	1.20	0.79	1.80	0.3911
Orofacial cleft	4.36	0.64	29.95	0.1342	1.62	0.21	12.48	0.6433	1.66	0.14	19.23	0.6841	0.38	0.04	3.41	0.3885
Vesicoureteral reflux	0.87	0.33	2.24	0.7665	0.32	0.14	0.75	0.0086	1.55	0.71	3.42	0.2749	1.79	0.65	4.97	0.2614
ADHD	2.81	0.72	10.95	0.1369	0.99	0.18	5.42	0.9922	1.04	0.19	5.64	0.9604	0.37	0.06	2.26	0.2821
Developmental delay	2.21	1.29	3.80	0.0040*	1.26	0.73	2.18	0.4005	1.29	0.72	2.32	0.3930	0.58	0.31	1.11	0.1013
Leukemia	--	--	--	0.9595	--	--	--	1.0000	--	--	--	0.9746	--	--	--	0.9747
Melanoma	--	--	--	1.0000	--	--	--	1.0000	--	--	--	1.0000	--	--	--	1.0000
malignant neoplasms of skin	--	--	--	1.0000	--	--	--	1.0000	--	--	--	1.0000	--	--	--	1.0000

**Minor disease**	**HR**	**(95% CI)**		***P-value***	**HR**	**(95% CI)**		***P-value***	**HR**	**(95% CI)**		***P-value***	**HR**	**(95% CI)**		***P-value***
Otitis media	1.75	1.11	2.77	0.0166	0.77	0.49	1.20	0.2481	1.41	0.89	2.23	0.1471	0.80	0.48	1.35	0.4102
Torticollis	1.67	0.57	4.89	0.3511	0.47	0.20	1.08	0.0760	2.71	1.05	6.99	0.0398	1.62	0.56	4.63	0.3725
Heart murmur	0.71	0.32	1.58	0.4030	1.57	0.70	3.55	0.2744	0.60	0.26	1.38	0.2337	0.85	0.31	2.36	0.7521
Newborn respiratory distress	1.59	0.91	2.79	0.1035	0.75	0.46	1.22	0.2415	1.96	1.12	3.41	0.0176	1.23	0.68	2.23	0.4974
Recurrent upper respiratory infection	1.11	1.01	1.22	0.0238	1.13	1.03	1.24	0.0107	0.94	0.85	1.03	0.1993	0.84	0.75	0.95	0.0033*
Croup (obstructive laryngitis)	1.18	0.83	1.67	0.3638	1.92	1.32	2.79	0.0007*	0.74	0.50	1.10	0.1331	0.63	0.40	0.99	0.0452
Colic	2.54	0.91	7.12	0.0759	2.54	0.44	14.83	0.2994	0.46	0.08	2.84	0.4052	0.18	0.03	1.13	0.0668
Jaundice	1.05	0.48	2.32	0.8992	1.10	0.54	2.23	0.7929	1.25	0.58	2.72	0.5698	1.19	0.48	2.94	0.7072
Urinary tract infection	0.13	0.02	1.07	0.0575	0.36	0.13	0.97	0.0422	1.05	0.43	2.53	0.9205	8.19	0.91	73.46	0.0604
Pyelonephritis	4.95	0.05	456.66	0.4883	0.48	0.05	5.06	0.5441	9.70	0.15	635.07	0.2871	1.96	0.09	45.23	0.6748
Atopic dermatitis	1.12	0.88	1.43	0.3735	1.57	1.20	2.07	0.0011*	0.71	0.54	0.94	0.0170	0.64	0.46	0.88	0.0058
Asthma	1.32	0.96	1.81	0.0872	1.40	1.04	1.89	0.0252	1.05	0.77	1.44	0.7554	0.80	0.55	1.17	0.2424
Eczema and dermatitis	1.04	0.90	1.22	0.5844	0.95	0.83	1.10	0.5108	1.12	0.97	1.30	0.1169	1.08	0.90	1.29	0.4142

*p<0.005.

Red text represents hazard ratios (HR) greater than 1 with statistical significance, while blue text indicates HRs less than 1 with statistical significance.

Comparing the four different embryo transfer methods and stages (**Table 4**), large for gestational age (LGA) outcomes were more likely in frozen embryo transfers compared to fresh transfers, with ORs of 1.669 for frozen cleavage stage versus fresh cleavage stage and 1.678 for frozen blastocyst versus fresh blastocyst transfers. Frozen embryo transfers generally exhibit safety profiles similar to those of fresh embryo transfers, with blastocyst transfers showing comparable outcomes to cleavage stage transfers for major diseases. The only significant finding was an elevated HR for developmental delay (HR 2.212, 95% CI 1.29-3.8, p-value 0.0040) in children conceived via fresh blastocyst transfers compared to fresh cleavage stage transfers. In terms of minor diseases, frozen blastocyst transfers were associated with a higher incidence of atopic dermatitis (HR 1.57, 95% CI 1.20-2.07, p-value 0.0011) and croup (HR 1.92, 95% CI 1.32- 2.79, p-value 0.0007) compared to frozen cleavage stage transfers. Furthermore, when comparing frozen and fresh embryo transfers, frozen transfers were linked to a reduced rate of respiratory diseases (OR 0.84, 95% CI 0.75-0.95, p-value 0.0011). Overall, no significant differences were observed in the incidence of major or minor diseases across the different embryo transfer methods and stages.

## Discussion

This study contributes to the growing body of literature (16–21) and recent systematic reviews (22, 23) on health outcomes in children conceived through assisted reproductive technologies, highlighting both the benefits and potential risks associated with various embryo transfer methods. The “freeze-all” strategy has been linked to reduced odds of ovarian hyperstimulation syndrome, with pregnancy and neonatal outcomes comparable to conventional methods (14, 20, 24–26). Furthermore, extending *in-vitro* culture to the blastocyst stage has been found to increase live birth rates, especially in patients with a favorable prognosis (27–29). While these approaches have demonstrated some advantages, their benefit has recently been called into question. A major limitation in previous studies has been the inability to simultaneously assess both the developmental stage at embryo transfer and the effects of cryopreservation. Our aim is to explore whether embryo handling procedures, including cryopreservation and stage of development, impact children’s health outcomes.

Our findings on neonatal outcomes align with prior research, indicating that transferring frozen-thawed embryos is associated with an increased risk of large-for-gestational-age (LGA) infants and cesarean sections, compared to fresh embryo transfers (14, 24, 30–32). All ART methods were associated with higher odds of preterm labor compared to natural conception. The increased preterm risk in ART cycles may be due to synchronization issues between the endometrium and embryos, as well as hormonal influences and inflammatory effects from continuous ovarian stimulation (33). This heightened risk of preterm birth and low birth weight may have implications for the long-term health of ART- conceived children.

The potential long-term consequences of high birth weight and LGA extend beyond infancy, potentially leading to obesity, diabetes, and cardiovascular disease later in life (34, 35). Recent studies from Finland have shown that boys born via frozen embryo transfer (FET) were heavier, with higher BMI and increased odds of being overweight compared to those born through fresh embryo transfer (ET) (36). Nevertheless, a 2023 systematic review from Italy found no significant differences between frozen and fresh transfers in terms of congenital malformations, neurodevelopmental disorders, growth, or chronic diseases (37). Similarly, another 2023 systematic review and network meta- analysis from Greece found no difference in the risk of congenital anomalies or adverse perinatal outcomes between blastocyst and cleavage stage transfers (22). Our study corroborates these findings, concluding that frozen and fresh embryo transfers, whether at the cleavage stage or blastocyst stage, result in comparable health outcomes in children.

Comparing natural conception, our investigation revealed that ART is associated with an increased risk of several conditions, most notably ADHD, particularly among children conceived through fresh blastocyst transfers. The neurodevelopmental risks of ICSI have been previously documented (4, 6), with potential mechanisms involving oxidative stress and DNA damage in selected sperm, or stress induced by procedural factors such as temperature, gas concentration, and pH value. The etiology of ADHD involves abnormalities in brain structure and function, as well as genetic influences, though the precise mechanisms remain unclear (38). Preterm birth has been established as a significant risk factor for ADHD (39, 40), and our study corroborates the association between ART procedures and increased rates of preterm birth and low birth weight. These findings highlight the importance of optimal perinatal care and early childhood interventions for children conceived through ART. Similarly, developmental delay was observed elevated in ART, which may be linked to multiple pregnancies and infertility-related factors, such as advanced parental age, regardless of ART use (41). The significant finding of increased developmental delay in children conceived via fresh blastocyst transfers compared to fresh cleavage-stage transfers may be partly attributed to the limited sample size. Notably, this elevated HR was not observed in frozen embryo transfers. The underlying mechanisms remain unclear, warranting further research to validate and clarify these findings.

Most large observational studies report a similar risk of cancer in children born after ART compared to the general population. However, a Danish study with a mean follow-up of 11.3 years found an elevated risk of childhood cancer, particularly leukemia, associated with the use of frozen embryo transfer (FET) (42). Similarly, a Nordic study identified an increased risk of epithelial tumors and melanoma following ART, as well as a higher risk of leukemia after FET (5). Freezing procedures may affect the embryonic cytoskeleton, DNA integrity, and the miRNA transcriptome (43–45). In the present study, the number of children aged 2–5 years with skin neoplasms, melanoma, and leukemia was limited, and no increased hazard ratio was observed. The conflicting results may partly be attributed to the low number of events in these disease and studies.

In our study, ART-conceived children exhibited higher rates of both major and minor diseases compared to those conceived naturally. However, specific ART techniques, including frozen embryo transfer and extended embryo culture, seem to have a limited impact on these outcomes. This may be explained by the underlying effect of parental infertility. A 2023 study from Australia supports this hypothesis, noting that the additional risk of congenital abnormalities was reduced and no longer statistically significant when comparing ART-conceived children to naturally conceived children born to parents with a history of infertility (46). This suggests that the increased risks observed in ART-conceived children may be partly explained by underlying parental infertility rather than ART procedures themselves.

In summary, while ART-conceived children face elevated risks for certain health conditions, particularly preterm birth and ADHD, the choice between frozen and fresh transfers or cleavage stage and blastocyst stage transfers does not appear to substantially influence these outcomes. These findings reinforce the importance of optimizing ART protocols and perinatal care, while also acknowledging the role of parental infertility in shaping the health of ART- conceived children.

### Strengths and limitations

This study has notable strengths and limitations that warrant consideration. A primary limitation is the inclusion of only children born after 20 weeks of gestation, thereby excluding data on spontaneous pregnancy loss and terminated pregnancies due to insufficient available information. As an observational study, our findings are subject to inherent biases, and causal inferences cannot be definitively established. Although we adjusted for several potential confounders, the impact of certain preexisting maternal and paternal comorbidities may not have been fully accounted for, potentially influencing the observed health outcomes in ART-conceived children. Additionally, due to the limited number of cases, we were unable to calculate reliable hazard ratios for rare conditions such as skin cancer and leukemia.

Moreover, specific clinical details that may influence outcomes were not captured in the dataset. For example, in frozen embryo transfer (FET) cycles, the type of endometrial preparation protocol—hormone replacement therapy (HRT) versus natural cycle—has been associated with differing perinatal risks in previous studies, but such information was unavailable in our data. Similarly, we could not distinguish between true singleton pregnancies and singletons resulting from twin pregnancies with vanishing twins, which may carry different risks for outcomes like preterm birth or low birth weight. Future research that integrates more detailed clinical and embryological information will be essential to further clarify these findings.

Despite these limitations, the study offers significant strengths. One key advantage is the concurrent assessment of both cryopreservation and the stage of embryo transfer, which provides clinically valuable insights. Furthermore, the use of a national cohort drawn from extensive registry data ensures a comprehensive and representative dataset for Taiwan. This robust dataset enhances the validity and generalizability of our findings, allowing for meaningful conclusions on ART outcomes at a population level.

## Conclusion

This study underscores notable differences in health outcomes between fresh and frozen embryo transfers and between ART and natural conception. Compared to natural conception, ART is associated with higher risks, particularly for preterm birth, ADHD, and developmental delay. However, while ART-related risks may partly stem from underlying parental infertility, the overall effects of procedures such as extended culture or cryopreservation on neonatal outcomes appear minimal. Notably, the use of frozen embryos or extended culture does not seem to amplify these risks. These insights are valuable for guiding patient counseling for individuals considering ART.

## Data Availability

The original contributions presented in the study are included in the article/supplementary material. Further inquiries can be directed to the corresponding author.
